# Differential reprogramming of breast cancer subtypes in 3D cultures and implications for sensitivity to targeted therapy

**DOI:** 10.1038/s41598-021-86664-7

**Published:** 2021-03-31

**Authors:** Esmee Koedoot, Liesanne Wolters, Marcel Smid, Peter Stoilov, Gerhard A. Burger, Bram Herpers, Kuan Yan, Leo S. Price, John W. M. Martens, Sylvia E. Le Dévédec, Bob van de Water

**Affiliations:** 1 Division of Drug Discovery and Safety, Leiden Academic Centre for Drug Research (LACDR), Leiden University, 2300 RA Leiden, The Netherlands; 2 Department of Medical Oncology and Cancer Genomics Netherlands, Erasmus MC Cancer Institute, Erasmus University Medical Center, Rotterdam, The Netherlands; 3 Department of Biochemistry and Cancer Institute, Robert C. Byrd Health Sciences Center, West Virginia University, Morgantown, USA; 4 OcellO, Leiden, The Netherlands

**Keywords:** Cancer, Breast cancer, Cell signalling, Cancer genomics

## Abstract

Screening for effective candidate drugs for breast cancer has shifted from two-dimensional (2D) to three-dimensional (3D) cultures. Here we systematically compared the transcriptomes of these different culture conditions by RNAseq of 14 BC cell lines cultured in both 2D and 3D conditions. All 3D BC cell cultures demonstrated increased mitochondrial metabolism and downregulated cell cycle programs. Luminal BC cells in 3D demonstrated overall limited reprogramming. 3D basal B BC cells showed increased expression of extracellular matrix (ECM) interaction genes, which coincides with an invasive phenotype not observed in other BC cells. Genes downregulated in 3D were associated with metastatic disease progression in BC patients, including cyclin dependent kinases and aurora kinases. Furthermore, the overall correlation of the cell line transcriptome to the BC patient transcriptome was increased in 3D cultures for all TNBC cell lines. To define the most optimal culture conditions to study the oncogenic pathway of interest, an open source bioinformatics strategy was established.

## Introduction

Breast cancer is the most prevalent cancer and the second leading cause of cancer death in women with an estimated 40,610 deaths in the United States in 2017^1^. Based on levels of the estrogen, progesterone and HER2 receptors, breast cancer can be divided in different subtypes. The triple-negative subtype (TNBC) lacking the expression of these three hormone receptors accounts for 15–20% of all tumors^2^ and is the most aggressive subtype, often leading to metastases^3,4^. Despite the efforts, there is still no targeted therapy for TNBC available^5^. A major reason for this lack in clinical translation may be the use of two-dimensional in vitro experiments that do poorly represent the three-dimensional (3D) tissue physiology observed in human cancer patients. To increase translation from in vitro findings to a clinical setting, different 3D culture systems are now explored, such as organoid cultures, patient-derived xenograft models, reprogrammed stem cell like models, tumor-on-a-chip and 3D cultures of immortalized breast cancer cell lines^6^. While the majority of breast cancer drug screening studies in the last decade have still been performed in 2D^7–12^, there is an increasing number of drug screens performed in more complex models such as patient-derived organoids^13,14^, tumor-on-a-chip^15^ and patient-derived xenograft^16^ models. Although these complex models better represent human physiology and should increase clinical translation^17–19^, drawbacks of these models include reduced reproducibility^17,20,21^, increasing costs, inconvenient maintenance, difficulties in expanding them and generating genetic modifications, making these models less suitable for high-throughput screening^22^. Next to the already widely studied phenotypic changes between different culturing models^16,23^ and phenotypic classification of different tumor subtypes^6^, transcriptomic and proteomic analyses can contribute to the understanding of the differences between established in vitro models and help to determine the most suitable model in terms of both clinical translation, costs and efficiency.

Here, we performed RNA-sequencing of 14 breast cancer cell lines cultured on a 2D plastic substrate as well as in a 3D matrigel-collagen environment. In this 3D model, cells spontaneously form spheroid-like structures exhibiting cell–cell as well as cell–extracellular matrix interactions, thereby changing their cell polarity and shape. We unraveled the transcriptomic differences linked to the invasive phenotype of basal B (or claudin-low) TNBC compared to basal A and luminal breast cancer and uncovered a spectrum of genes higher expressed in 2D cultures that were related to metastatic progression in breast cancer patients. Since the transcriptomic correlation of in vitro cultured cell models to patient tumor tissue was highly subtype and pathway dependent, we established a bioinformatics tool that can be used in future studies to select the most suitable cell type and culture conditions for the pathway of interest. Altogether, this study unraveled the transcriptomic variance between different breast cancer in vitro models and provides an important database that can contribute to selection of the most effective and relevant drug candidates for the treatment of TNBC.

## Results

### mRNA profiling of breast cancer cells cultured in 3D revealed downregulation of cell cycle-related genes and upregulation of mitochondrial genes

**Figure 1:**
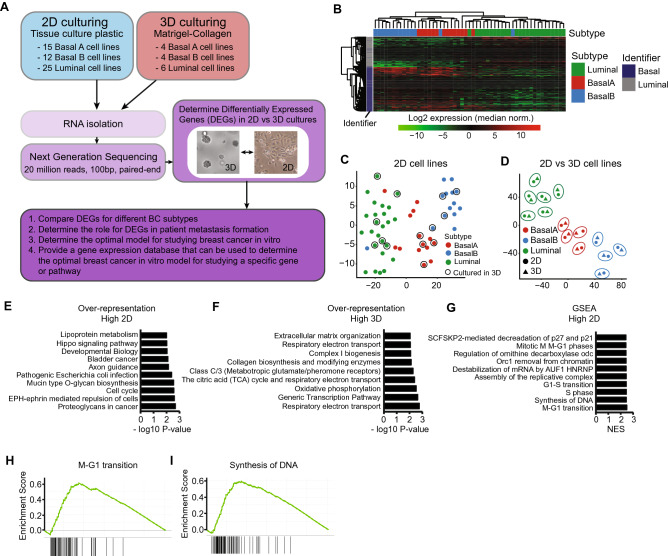
RNA sequencing of breast cancer cell lines cultured in 2D and 3D environments. (**A**) Experimental overview. RNA was isolated from cell lines cultured in 2D and 3D conditions (see Methods section for more details), upon which next generation sequencing was performed. Cell line specific Differentially Expressed Genes (DEGs) were selected based on log2 fold change > 1 or <  − 1. Subtype specific DEGs were determined using a paired cell line analysis and selected if the parametric *p*-value < 0.01. See “Methods” section for further details. (**B**) Hierarchical clustering (complete linkage, correlation) of log2 expression levels of previously defined basal and luminal identifiers^29^ in 2D cultured cell lines. For every gene, expression levels were normalized to the median cell line expression. (**C**) T-sne of all cell lines cultured in 2D based on log2 normalized reads from all detected genes. Encircled cell lines were also cultured in 3D in this study. (**D**) T-sne of cell lines cultured in both 2D and 3D conditions based on log2 normalized reads from all detected genes. 2D and 3D conditions of the same cell line are encircled. (**E**) Top 10 over-represented pathways in DEGs higher expressed in 2D cultures. DEGs were determined using paired analysis for all cell lines, *p* < 0.01. (**F**) Top 10 over-represented pathways in DEGs higher expressed in 3D cultures. DEGs were determined using paired analysis for all cell lines, *p* < 0.01. (**G**) Top 10 enriched pathways higher expressed in 2D cultures using a ranked gene set enrichment analysis (GSEA). (**H**) and (**I**) Examples of GSEA of pathways enriched in 2D cultures. The enrichment score (ES) represents the degree to which a pathway is overrepresented at the top or bottom of the gene ranking (add ref Subramanian, PNAS, 2005).

To understand how cell culture systems affect the transcriptome of breast cancer (BC) cells, we performed RNA sequencing of 52 human breast cancer cell lines cultured on 2D tissue culture plastic and 14 cell lines cultured in a 3D matrigel-collagen environment (Fig. 1A, Suppl. Table 1). The selection of the 14 cell lines was based on previously defined subtype classifications^24–28^ with selected cell lines representing the different BC subtypes (luminal, basal A and basal B (often named claudin-low)). These cell line subtypes were validated in our RNA sequencing dataset; hierarchical clustering based on RNA expression of previously published luminal and basal markers clearly separated the different subtypes (Fig. 1B and Suppl. Fig. 1, Suppl. Table 2)^24,29^. Moreover, cell lines could also be assigned to these subtypes based on whole transcriptomic differences (Fig. 1C). Three cell lines (SUM149PT, SUM225CWN and HCC1500) did not cluster with their assigned subtype (Fig. 1B), which was in agreement with the ambiguous subtype classification described in the literature (Suppl. Table 3)^24–28^. To visualize the global transcriptomic differences between 2 and 3D culturing systems, we created a T-sne plot using RNA expression levels from the cell lines that were cultured both in 2D and 3D culture conditions (Fig. 1D). Cell lines separated consistently based on their subtype and, interestingly, the differences between cell lines were much larger than the differences between 2D/3D culture conditions; 2D and 3D samples of the same cell line clustered together (Fig. 1D, encircled). However, these 2D and 3D samples were still clearly separated, revealing significant changes in gene expression patterns between the different culture conditions. To investigate the altered pathways comparing 2D to 3D culturing conditions, we calculated the significantly differentially expressed genes (DEGs) pairing 2D and 3D samples for every cell line, followed by over-representation analysis (Fig. 1E/F) and ranked gene set enrichment analysis (GSEA) (Fig. 1G). In total, we identified 452 genes higher expressed in 2D and 1762 genes higher expressed in 3D (Suppl. Table 4). Over-represented and enriched pathways in 2D cultures include mainly cell cycle and cancer related pathways (Fig. 1E/G/H/I), while pathways related to oxidative phosphorylation and transcription were higher expressed in 3D systems.

### 3D culturing induced expression of extracellular matrix organization genes in Basal B cell lines

**Figure 2:**
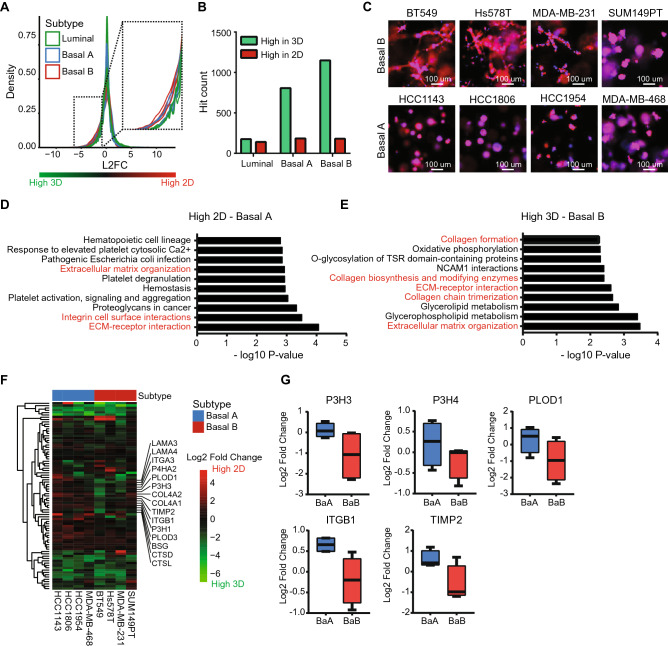
Subtype specific differences in 2D and 3D breast cancer cultures. (**A**) Density plot of log2 fold change in gene expression comparing 2D to 3D cultures of 14 cell lines (4 basal A, 4 basal B and 6 luminal). (**B**) Significantly DEG count for different subtypes based on paired analysis and *p* < 0.01. (**C**) Cellular morphology of basal A and basal B cell lines cultured in 3D. (**D**) Top 10 over-represented pathways in the high 2D DEGs in basal A cell lines. Migration and invasion related pathways are highlighted in red. DEGs were determined by paired analysis of basal A cell lines, *p* < 0.01. (**E**) Top 10 over-represented pathways in the high 3D DEGs in basal B cell lines. Migration and invasion related pathways are highlighted in red. DEGs were determined by paired analysis of basal B cell lines, *p* < 0.01. (**F**) Heatmap (complete linkage, Euclidean distance) of log2 fold change in expression of genes in the extracellular matrix organization pathway comparing 2D to 3D cultures. (**G**) Log2 fold change of expression levels of genes in the extracellular matrix pathway comparing 2D to 3D cultures in basal A and basal B cell lines. Selected genes were higher expressed in 2D in the basal A cell lines, but higher expressed in 3D in the basal B cell lines. Log2 fold change was determined using a paired analysis per subtype.

Previously, we showed that gene expression differences between 2 and 3D cultures can be subtype and cell line specific. Therefore, we now examined the DEGs for each cell line. On average, ~ 3000 genes were higher expressed in 2D per cell line (log2 fold change > 1) (Suppl. Fig. 2A), independent of cell line or subtype. Interestingly, in 3D cultures the number of DEGs was higher in basal compared to luminal cell lines (~ 2000 vs. 4500; Fig. 2A and Suppl. Fig. 2B). This was confirmed by the number of significantly DEGs per subtype: for the luminal subtype ~ 200 genes were higher expressed in both 2D and 3D, while for the basal subtype also ~ 200 genes were higher expressed in 2D, but ~ 800 (basal A) and ~ 1100 (basal B) genes were upregulated in 3D (Fig. 2B). Furthermore, the majority of the DEGs for the basal subtypes were overlapping (Suppl. Fig. 2C). Interestingly, while in 2D cultures both basal A and basal B subtypes displayed an active migratory behavior (Suppl. Fig. 3), in 3D cultures only basal B showed an invasive phenotype (Fig. 2C). Similar morphological differences were previously observed by us and others when cell lines were cultured on top of a 3D laminin-rich extracellular matrix composed of matrigel and bone marrow extract^30^. In order to identify the underlying pathways that contribute to this differential invasive capacity, we performed an over-representation analysis for the subtype-specific DEGs in basal A and basal B cell lines (Fig. 2D/E, Suppl. Fig. 2D/E). Remarkably, pathways related to cell–matrix interactions, extracellular matrix organization, collagen synthesis and integrin cell surface interactions were higher expressed in 2D for the basal A subtype (Fig. 2D), while higher expressed in 3D for the basal B subtype (Fig. 2E). Only a small subset of the extracellular matrix organization pathway was upregulated in 3D settings in basal B cell lines, while equally expressed or down-regulated in basal A cells (Fig. 2F). Interestingly, this subset includes the genes PLOD1, P3H3 and P3H4, which together form a complex that catalyzes hydroxylation of lysine residues in collagen chains and is required for proper collagen biosynthesis and modification^31^ (Fig. 2G). Other interesting members identified in this clusters are genes involved in cell adhesion, such as ITGB1 (Fig. 2G) and genes involved in breakdown of the extracellular matrix such as TIMP2 (Fig. 2G).

### Metastatic breast cancer genes are downregulated in 3D cultures

**Figure 3:**
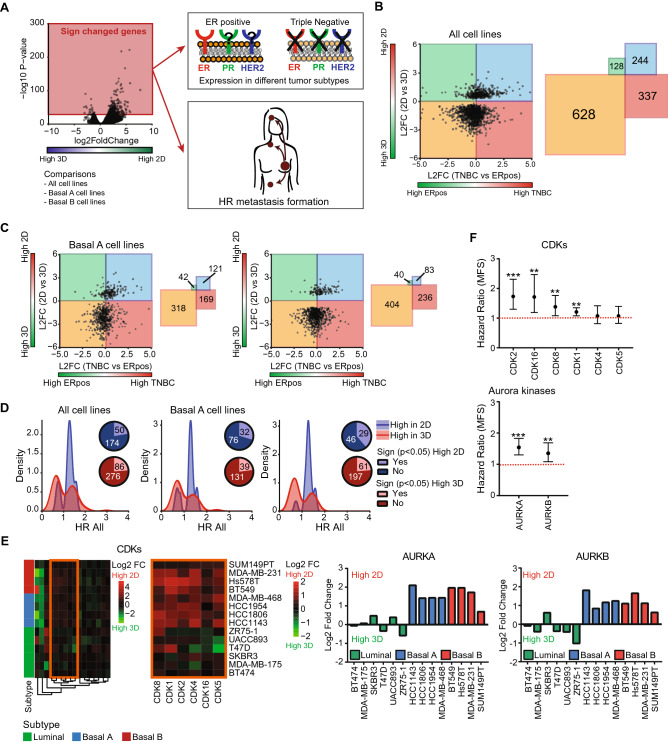
Role of DEGs in breast cancer aggressiveness and metastasis formation. (**A**) Significantly DEGs comparing all cell lines, basal A cell lines or basal B cell lines were selected using a paired cell line analysis per subtype, *p* < 0.01. For these genes, the expression in different tumor subtypes as well as the relation to metastasis formation was determined. (**B**) Log2 fold change of DEGs (paired analysis of all cell lines, *p* < 0.01) comparing 2D to 3D cell cultures versus log2 fold change comparing TNBC to ER positive primary tumors (left). Number of genes in each quadrant (right). (**C**) Log2 fold change of DEGs (paired analysis of basal A and basal B cells, *p* < 0.01) comparing 2D to 3D cell cultures versus log2 fold change comparing TNBC to ER positive breast tumors (left). Number of genes in each quadrant (right). (**D**) Density plot of DEGs (paired analysis of all, basal A and basal B cell lines respectively, *p* < 0.01) with a significant hazard ratio (HR). Blue = density plot for DEGs with increased expression in 2D cultures. Red = density plot for DEGs with increased expression in 3D cultures. Venn diagrams show the fraction of DEGs showing a significant HR. (**E**) Heatmap (Euclidean distance, complete linkage) of log2 fold change of CDK expression levels comparing 2D to 3D cultures (orange cluster is enlarged on the right) and log2 fold change of aurora kinase A and B comparing 2D to 3D cultures for different cell lines. (**F**) Hazard ratios and 95% confidence interval (CI) for CDKs higher expressed in 2D and aurora kinase A and B.

Our analysis of the differential mRNA profiles between 2 and 3D cultures provided us with new insights on the transcriptional reprogramming related to basal cell line invasiveness in 3D cultures. Next, we wondered whether these newly identified DEGs would also play a role in breast cancer progression and metastasis formation in patients. Therefore, we examined the expression of DEGs in the different primary breast tumor subtypes using RNA sequencing data from 923 primary breast tumors (116 TNBC and 807 ER positive (of which 682 PR positive) tumors) derived from The Cancer Genome Atlas (TCGA). Since we observed clear differences in culture-specific gene expression changes for the different subtypes, we performed these analyses for the DEGs obtained from the paired analysis for all cell lines, basal A and basal B specific DEGs (Fig. 3A). Independent of the cell line subtype, DEGs higher expressed in 3D were higher expressed in the less aggressive ER positive subtype, while DEGs higher expressed in 2D were higher expressed in the more aggressive TNBC subtype (Fig. 3B,C). To investigate if these DEGs are also associated with metastatic progression in breast cancer patients, the hazard ratios (HRs) for distant metastasis-free survival (DMFS) were examined using microarray data of 867 untreated lymph-node negative breast cancer patients (Fig. 3A). DEGs with a significant HR were selected and the distribution of these HRs was plotted (Fig. 3D). DEGs higher expressed in 3D showed slightly more DEGs of which high expression is negatively related to metastatic progression in all subtypes (HR < 1). Interestingly, DEGs higher expressed in 2D were mainly positively related to metastatic progression (HR > 1) independent of the subtype. Examples of such DEGs down-regulated in 3D cultures and significantly related to metastatic progression were cyclin dependent kinases (CDKs), aurora kinases (Fig. 3E,F) and proteasomal factors (Suppl. Fig. 4A-B). Since dysregulation of cell cycle is one of the hallmarks of cancer^32^, CDKs and aurora kinases are attractive targets for new cancer therapy^33–36^. Moreover, cancer cells might be more dependent on the proteasome for the elimination of abnormal proteins due to high proliferation rate and genetic instability^37,38^. Altogether, DEGs higher expressed in 2D cultures were also higher expressed in the more aggressive TNBC subtype and positively associated with metastatic progression.

### Differential sensitivity of 3D TNBC cultures to clinical relevant kinase inhibitors

**Figure 4:**
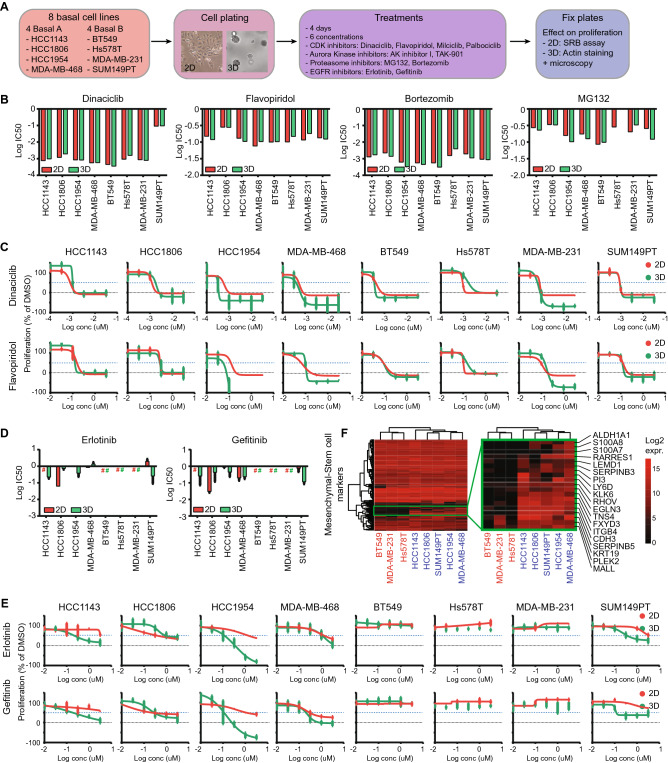
Effect of DEG inhibitors on cell line proliferation in 2D and 3D. (**A**) Experimental setup. 4 basal A and 4 basal B cell lines were plated in 2D and 3D conditions, after which they were treated with 6 different concentrations of CDK, Aurora Kinase, Proteasome or EGFR inhibitors. 4 days after treatment, cells were fixed and proliferation was assessed. (**B**) Log IC50 (uM) for CDK inhibitors dinaciclib en flavopiridol and proteasome inhibitors bortezomib and MG132 in 2D and 3D cultures of basal A and basal B cell lines. (**C**) Dose response curves of dinaciclib en flavopiridol in 2D and 3D cultures of basal A and basal B cell lines. Mean + stdev for technical duplicates in 2D cultures. Mean + stdev for technical quadruplicates in 3D cultures. (**D**) Log IC50 of EGFR inhibitors erlotinib and gefitinib in basal A and basal B cell lines. (**E**) Dose–response curves of erlotinib and gefitinib in 2D and 3D cultures of basal A and basal B cell lines. Mean + stdev for technical duplicates in 2D cultures. Mean + stdev for technical quadruplicates in 3D cultures. (**F**) Heatmap (complete linkage, Euclidean distance) of log2 expression of mesenchymal-stem cell markers in basal A and basal B cell lines. Cell line color indicates 3D invasiveness; red = invasive, blue = non-invasive.

CDKs, aurora kinases and proteasomal inhibitors are critical candidate drugs some of which are already in clinical trials or application; these inhibitors have mainly been studied in 2D cell culture systems^39–42^. The respective targets of these various inhibitors were significantly lower expressed in 3D cultures (Fig. 3E, Suppl. Fig. 4A), potentially leading to a change in sensitivity. Here, we tested whether BC cell line drug sensitivity is dependent on the differential transcriptome driven by the 2D and 3D culturing conditions. 4 basal A and 4 basal B breast cancer cell lines were cultured in both culturing systems, treated with several CDK inhibitors with different CDK selectivity (dinaciclib, flavopiridol, milciclib or palbociclib, Suppl. Table 5), aurora kinase inhibitors (aurora Kinase Inhibitor I or TAK-901), proteasome inhibitors (MG132 or bortezomib) or EGFR inhibitors (erlotinib and gefitinib), after which the effect on proliferation was quantified using image-based analysis (Fig. 4A). Although the growth rates differed for the eight TNBC cell lines, there were neither subtype specific proliferation differences in either 2D or 3D cultures (Suppl. Fig. 5A-D) nor a correlation between growth rates in 2D and 3D cultures (Suppl. Fig. 5E). As expected by the decrease in expression of cell cycle related genes, all cell lines showed a significant decrease in growth rate in 3D cultures compared to 2D cultures (Suppl. Fig. 5F-G). Remarkably, this differential growth rate between 2 and 3D did not result in a significant change in sensitivity to anti-proliferative treatments such as CDK and aurora kinase inhibitors (Fig. 4B-C, Suppl. Figure 6A-C). The same was true for the proteasome; although the expression of many proteasome factors was down-regulated in 3D, the sensitivity towards proteasome inhibitors MG132 and bortezomib did not change (Fig. 4B, Suppl. Figure 6D).

EGFR is higher expressed in TNBC compared to ER positive BC and is another important drug target in BC treatment^43–45^. In contrast to CDKs, aurora kinases and proteasomal factors, EGFR was higher expressed in 3D for some basal cell lines (Suppl. Figure 7A). Therefore we predicted that these cells would be more sensitive to EGFR inhibitors such as erlotinib and gefitinib. The majority of the basal cell lines used in this study were resistant to EGFR inhibitor treatment in 2D, except for MDA-MB-468 and a partial sensitivity seen with HCC1806 (Fig. 4D,E). Interestingly, HCC1143, HCC1954 and SUM149PT cell lines became more sensitive to both erlotinib and gefitinib in 3D cultures, while BT549, Hs578T and MDA-MB-231 remained resistant. This increased sensitivity was likely not directly related to EGFR RNA expression or its signaling pathway (Suppl. Figure 7A), since EGFR expression was also almost twice upregulated in the resistant MDA-MB-231 cell line. As shown before, basal cell lines in 3D cultures demonstrated differences in invasive behavior with a significantly higher invasion capacity for basal B (except for SUM149PT) compared to basal A cell lines (Fig. 2C, Suppl. Figure 7B-C). Interestingly, only the invasive cell lines remained resistant to EGFR inhibitors in 3D cultures. This was not related to differences in 3D growth rate (Suppl. Figure 7D), EGFR expression (Suppl. Figure 7E-F), expression of genes involved in EGFR signaling (Suppl. Figure 7G-H) or mutations in the EGFR gene based on the COSMIC database (data not shown). A recent paper by *Savage *et al.observed heterogeneous EGFR expression in breast cancer patient-derived xenografts (PDX) and showed that the sensitivity to EGFR inhibitors such as gefitinib is determined by the variation rather than the mean EGFR expression; PDX models bearing heterogeneous expression of EGFR were more sensitive towards gefitinib^46^. EGFR-high subpopulations were characterized by growth inhibition upon gefitinib treatment and enhanced expression of mesenchymal-stem cell markers^46^. Interestingly, the same mesenchymal-stem cell markers were also more highly expressed in non-invasive EGFR-inhibitor sensitive cells in both 2D (Fig. 4F) and 3D cultures. We hypothesize that when losing oncogenic proliferative signaling pathways in 3D conditions, these cell lines became more dependent on EGFR related stem cell-mesenchymal pathways and therefore more vulnerable to EGFR inhibitor treatment.

Altogether, we demonstrated that differential gene expression does not always relate to changes in drug sensitivity, especially in case of cell cycle related genes. Non-invasive cells were sensitive to EGFR inhibitors in 3D systems irrespective of the EGFR expression levels, suggesting that other factors than single gene expression patterns might be involved in drug sensitivity.

### Gene expression patterns of basal cell lines cultured in 3D are better correlated to human patient tumors than 2D cultured samples

**Figure 5:**
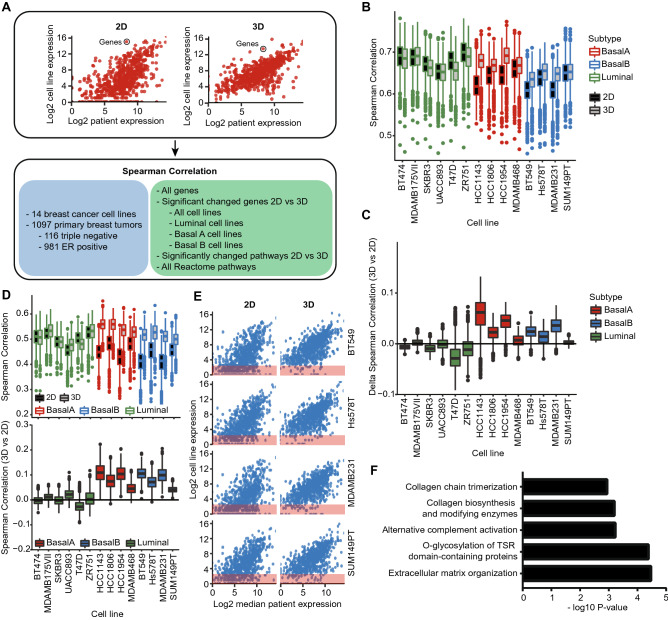
Comparison the transcriptomes of 2D and 3D cell culture models with patient derived primary tumor transcriptomes. (**A**) Analysis overview. Spearman correlations were calculated between the different transcriptomes using log2 normalized gene expression levels taking into consideration cell line subtypes, tumor subtypes and different gene sets. (**B**) Spearman correlation of log2 normalized RNA expression levels between cell lines and patient primary breast tumors (n = 1097). Boxes represent median and interquartile range of all tumor correlations. All genes and all tumors (irrespective of subtype) were included in the correlation calculations. (**C**) Difference in spearman correlation comparing 2D and 3D cell line cultures matched by patient tumor. All genes and all tumors were included in the correlation calculations. Boxes represent median and interquartile range of the difference in spearman correlation for all tumors. (**D**) Spearman correlation of log2 normalized RNA expression levels of basal B DEGs (paired analysis, *p* < 0.01) between cell lines and patient primary breast tumors (n = 1097). Boxes represent median and interquartile range of all tumor correlations. Only DEGs comparing 2D and 3D cultures for the basal B cell lines were included in the correlation calculations (top). Difference in spearman correlation comparing 2D and 3D cell line cultures matched by patient tumor. Only DEGs comparing 2D and 3D cultures for the basal B cell lines were included in the correlation calculations (bottom). Boxes represent median and interquartile range of the difference in spearman correlation for all tumors. (**E**) Log2 normalized RNA expression levels of basal B DEGs comparing 2D to 3D cultures (paired analysis, *p* < 0.01) in basal B cell lines and TCGA patient primary breast tumors. Red squares: genes with log2 RNA expression < 2. (**F**) Top 5 pathways of over-representation analysis of basal B DEGs comparing 2D to 3D cultures (paired analysis, *p* < 0.01) not or low expressed in 2D cultures and higher expressed in 3D cultures.

For efficient drug development, it is vital to choose the optimal in vitro test system in which the regulation of pathways correlates as close as possible to the human primary tumor situation. To compare the translational value of both 2D and 3D culture systems for human patient BC tumors, we calculated the correlation between cell line and patient RNA expression levels derived from TCGA (Fig. 5A). Correlations were calculated based on all genes and patients, but also focused on DEGs between 2 and 3D culturing systems, specific pathways or specific patient tumor subtypes (Fig. 5A). Taking into consideration all genes and all patient tumors, luminal cell lines in 2D culture were better correlated to patient tumors than basal cell lines in 2D culture (Fig. 5B). However, for the basal subtypes only, this correlation improved significantly for 3D cultures (Fig. 5B,C). The same trend was observed when only analyzing ER positive tumors both when taking into consideration all genes (Suppl. Figure 8A) and genes involved in the Reactome estrogen receptor-mediated signaling pathway (Suppl. Figure 8B). For TNBC, the correlation of basal and luminal cell lines was similar in 2D cultures, but much higher for basal (mainly basal A) cell lines in 3D cultures (Suppl. Figure 8C). Similarly, DEGs mainly contributed to increased correlations for the basal cell lines (Fig. 5D). Remarkably, the correlation of basal-specific DEGs showed a slightly larger increase in ER positive tumors compared to TNBC (Suppl. Figure 9 and Suppl. Figure 10) suggesting that the DEGs might have an important function in all patient tumors regardless of the subtype. Plotting the correlations of basal B subtype specific DEGs demonstrated that the correlation increase in 3D could be attributed to a small subset of DEGs; these genes were not or low expressed in 2D, but show increased levels in 3D (Fig. 5E, red box). Gene set enrichment analysis on this subset of genes revealed that the majority of these DEGs is involved in extracellular matrix organization and collagen formation (Fig. 5F), suggesting that studying these pathways in 3D cultures might be more relevant for human patient tumors. Furthermore, assessment of Reactome pathway expression correlations between all human patient tumors and 2D and 3D models emphasized that the optimal model is highly dependent on the pathway of interest (Suppl. Figure 11A).

## Discussion

Although there are increasing efforts to use 3D tumor cell cultures to evaluate the efficacy of candidate target therapies, a systematic comparison between the cellular physiology of 2D and 3D cultures is still lacking. Here we compared the transcriptome of BC cell lines cultured in 2D and 3D conditions to (1) uncover the differential pathway expression linked to subtype-specific morphological changes and (2) identify the most suitable model for future drug screens. Consistent with previous comparisons of 2D and 3D cultures^47^, we demonstrate that BC cells in a 3D matrigel environment have a decreased expression of genes important in cell cycle pathways, including various CDKs and aurora kinases, regardless of the subtype. Of relevance, despite the downregulation of these gene sets in 3D, we did not observe a differential potency of CDK and aurora kinase inhibitors comparing 2D and 3D cultures. Regardless of this lack of potency, the gene expression patterns of 3D cultures better resembled the BC patient transcriptome, in particular for TNBC subtypes.

We observed that cell lines of the basal A subtype showed a clear non-invasive spheroid morphology in 3D. In contrast, cell lines of the basal B subtype demonstrated a distinct invasive phenotype; an exception for the basal B phenotype was the SUM149PT, that shows ambiguous classification and has also sometimes been classified as basal A^25,26,28^. Also needs to be noted that the MDA-MB-231 cell line that was classified as basal B and has been used in this context in many studies, demonstrated to be a poor basal cell line representative in a recent study^48^. These contrasting phenotypes between basal A and basal B cells are likely related to differences in expression of a small subset of extracellular matrix interacting and cell–cell adhesion genes. Thus, the basal B cells upregulated genes involved in collagen biosynthesis and breakdown of the extracellular matrix in 3D cultures, while these genes were downregulated in basal A cells. Moreover, the higher basal levels of E-cadherin in the basal A subtype could contribute to the less invasive phenotype observed in 3D cultures. The differences in cell line invasiveness and related pathway regulation for basal subtypes could be specific to the selected culture environment. Future studies using a 3D environment without the presence of an external extracellular matrix such as ultra-low attachment plates or spinner vessels would reveal the effects of the matrix composition on pathway regulation and cell phenotype.

CDKs, aurora kinases and proteasomal factors that were higher expressed in 2D, showed genuine higher expression levels in more aggressive breast tumors and cell lines, and were positively related to metastatic disease in breast cancer patients. Several aurora kinase inhibitors and Cell cycle-related CDK inhibitors have been developed of which some show promising results in clinical trials and are almost facing towards the clinic^33–36^. Next, proteasomal inhibition could disrupt essential cellular pathways resulting in cancer cell death ^37,38,49,50^. To investigate whether transcriptomic differences change drug sensitivity and define the optimal in vitro model for future drug screening; we systematically treated cell lines in both 2D and 3D cultures with specific inhibitors against CDKs, aurora kinases and the proteasome. Whereas previous studies mainly identified reduced sensitivity towards established drugs (chemotherapeutics, endocrine agents and HER2-targeting agents) in 3D cultures in mostly HER2 positive cell lines^51–53^, no change in sensitivity for CDKs, aurora kinases and proteasomal factors comparing 2D and 3D cultures was observed with our experimental setup. While the drugs that we tested were not evaluated in these studies, the discrepancies can also be explained by the differences in cell lines used and culture conditions. Additionally, we cannot exclude that limited differential sensitivity between 2 and 3D for the selected drug targets is related to limited changes of the target protein levels. For example, EGFR expression per se does not explain EGFR inhibitor sensitivity, and that EGFR inhibitors are effective in blocking EGFR auto-phosphorylation, without impacting on downstream signaling^54^. Hence, the TNBC proliferative and survival signaling wiring is complex and multiple pathways can contribute to overcome kinase inhibitor efficacy. Regardless, our data do indicate that across a broader panel of TNBC cell lines, typically 2D screening for the candidate drug targets will be as effective as 3D screening in identifying drug responses. To determine the optimal future screening model to assess efficacy of new targeted therapies, a thorough understanding of the overall clinical translation of the test system for specific cancer types, including TNBC, will be essential.

For basal BC cell lines the transcriptome of 3D cultures demonstrated increased similarity towards human patient tumor transcriptomes, which was mainly caused by elevated levels of genes involved in extracellular matrix interactions. On the contrary, the transcriptome profile of luminal cell lines was already highly comparable to the patient tumor transcriptome in 2D conditions, but did not improve in 3D settings. Importantly, similarity of transcript levels was highly pathway and subtype dependent. Next to the pathway of interest, the ideal in vitro model might change depending on the BC subtype of interest. In general, pathways in triple-negative patient tumors are better correlated to basal cell lines than the same pathways in ER-positive tumors.

Altogether, we can conclude that our systematic transcriptome analysis uncovered major differences between 2 and 3D models thereby increasing the global understanding of these in vitro test systems. Defining ‘the best in vitro model’ with efficient clinical translation is yet difficult and dependent on both the pathway and subtype of interest, although in general 3D cultures correlated better to human patient tumors. To support such a selection, we established an in silico web tool in to allow (1) differential expression of genes between 2 and 3D BC cell lines, (2) collect gene expression levels in different breast cancer subtypes (cell lines as well as patient tumor data) and (3) calculate correlation between different cell lines/culture conditions with human patient tumors (10.5281/zenodo.4560297).

We performed a systematic transcriptomic comparison between 2 and 3D BC cell lines. Although clear transcriptomic differences were identified between 2 and 3D culturing systems, additional proteomic analysis would add a layer of information that could help to validate the transcriptomic findings and more intensively discriminate between the different models. In this study, we did not include other in vitro cell models that are of relevance for BC anticancer therapy development, including BC tumor organoids, short-term patient-derived xenograft cell cultures, stem cell reprogramming and tumors-on-a-chip^13,55–57^. These models that are closer to the patient situation have higher costs and cannot yet be applied in similar throughput as 2D plastic models, but are recently widely exploited for their application in drug screening^58–61^. Future systematic comparisons of the transcriptome and proteome of all these test systems in comparison with large BC patient transcriptome and proteome datasets, and their response to candidate drug targeted therapeutic approaches will be essential to provide clarity about the most optimal pre-clinical in vitro test systems.

## Materials and methods

### Cell culture

All cell lines were purchased from ATCC. Cells were grown in RPMI-1640 medium (Gibco, ThermoFisher Scientific, Breda, The Netherlands) supplemented with 10% FBS (GE Healthcare, Landsmeer, The Netherlands), 25 IU/ml penicillin and 25 µg/ml streptomycin (full RPMI) (ThermoFisher Scientific) at 37 °C in a humidified 5% CO_2_ incubator. For 2D cultures, cells were grown on plastic tissue culture plates (Corning). For 3D cultures, cells were grown in a gel-mixture containing 6 mg/ml matrigel (Corning, Lot# 5061003), 0.5 mg/ml collagen (Corning, Lot# 5092001), 3.7 g/L NaHCO_3_ and 0.05 M HEPES.

### RNA isolation

For 2D cultures, cells were plated in a 6-well tissue culture plate (Corning, 3516) in full RPMI. RNA was collected 2 days after plating at 70% confluency using RNeasy plus mini kit (Qiagen) according to the manufacturer’s protocol.

For 3D cultures, cells were plated in a 24-wells tissue culture plate (Corning, 3524). Wells were pre-coated with 200 μl matrigel (Corning, Lot# 5061003, 9.4 mg/ml) and cells were plated in a total volume of 400 μl. The following densities were used for the different cell lines: MDA-MB-231 (200 cells/μl), Hs578T (200 cells/μl), BT549 (200 cells/μl), SUM149PT (250 cells/μl), HCC1143 (250 cells/μl), HCC1806 (250 cells/μl), HCC1954 (250 cells/μl) and MDA-MB-468 (250 cells/μl). After 30 min incubation at 37 °C, 1200 μl full RPMI was added. RNA was collected 7 days after plating using the QIAzol method to reduce matrigel pollution. RPMI was removed, 1.2 ml QIAzol (Qiagen) was added per well and cells were lysed by repetitive pipetting. Cells were incubated for 5 min at room temperature and 240 μl chloroform was added, followed by 2–3 min incubation at room temperature. Samples were centrifuged for 15 min at 4 °C (12,000 × *g*), the upper aqueous phase was collected and RNA was precipitated by mixing with 600 μl isopropanol. Samples were incubated for 10 min at room temperature, followed by 15 min centrifugation at 4 °C (12,000 × *g*) and washing with 1.2 ml 75% v/v ethanol. Samples were centrifuged for 5 min at 4 °C (7500 × *g*). Then the supernatant was removed and the pellet was air-dried and dissolved in 30 μl RNase free water.

### RNA sequencing and analysis

DNA libraries were prepared with the TruSeq Stranded mRNA Library Prep Kit and sequenced according to the Illumina TruSeq v3 protocol on an Illumina HiSeq2500 sequencer. 100 base pair paired-end reads were generated and alignment was performed using the HiSat2 aligner (version 2.2.0.4) against the human GRCh38 reference genome. Gene expression was quantified using the HTseq-count software (version 0.6.1) based on the ENSEMBL gene annotation for GRCH38 (release 84). Count data was normalized using the DESeq2 package^62^ and DEGs were selected based on log2 fold change > 1 or <  − 1. Paired t-test were performed on the normalized RNA-seq data using BRB-ArrayTools (v4.4.1) developed by Dr. Richard Simon and the BRB-ArrayTools Development Team and R (v3.2.2). Genes with *p*-value < 0.01 were considered significant. Upon acceptance, RNA sequencing data will be available in the Sequence Read Archive.

### Drug screening

For 2D drug screening, cells were plated in a 96-well tissue culture plate (Corning, 3599) using the following cell densities: HCC1143 (8000 cells/well), HCC1806 (8,000 cells/well), HCC1954 (8000 cells/well), MDA-MB-468 (10,000 cells/well), Hs578T (4,000 cells/well), MDA-MB-231 (5000 cells/well), SUM149PT (8000 cells/well) and BT549 (8,000 cells/well). Drug treatment was performed 16 h after plating and 4 days after treatment, cells were fixed for sulforhodamine B (SRB) colorimetric proliferation assay^63^ previously described by our group^64^.

For 3D drug screening, cells were plated in a 384-well µclear plate (Greiner, 781091) using a CyBi Selma 96/60 robotic liquid dispenser (Analytik Jena AG, Jena, Germany). The same gel composition and cell densities were used as in the RNA isolation procedure. Cells were plated in a total volume of 14.5 μl matrigel-collagen mix. After 30 min incubation at 37 °C, 44.5 μl full RPMI was added. Drug treatment was performed 3 days after plating and 4 days after treatment, cells were fixed and stained in 0.05 µM Phalloidin Rhodamin (Sigma Aldrich), 0.4 µg/ml Hoechst 33258 (Fisher Biotech), 0.1% Triton X-100 Triton (Sigma Aldrich) and 3.7% formaldehyde (Sigma Aldrich) in PBS O/N at 4 °C. Plates were washed with PBS and imaged using ImageXpress Micro XLS system (Molecular Devices, CA, USA).

Both in 2D and 3D cultures, cells were treated with 6 concentrations of the following inhibitors: MG132, bortezomib, flavopiridol HCl, dinaciclib, palbociclib, milciclib, aurora kinase inhibitor I, TAK-901, gefitinib and erlotinib (all Selleck Chem). DMSO was used as drug vehicle control.

### Drug screen normalization and analysis

SRB absorbance values were used to analyze 2D cell line proliferation. To analyze the 3D cultures the acquired image stacks (4 × objective, 40 sections per well, 50 µm step size) were processed in OcellO Ominer 3D image analysis platform. The software processes the DAPI channel to measure the number of nuclei and the shapes of the nuclei in each image stack and integrates this information with the shape and number of tumor cell clusters detected in the TRITC channel. The software was used to process the total phalloidin area as measurement of proliferation.

Proliferation was normalized to the absorbance (2D) or phalloidin area (3D) before treatment (set at 0%) and DMSO treatment (set at 100%). A nonlinear sigmoidal dose–response (with variable slope) curve was used to fit the data and retrieve the IC50 values using Graphpad Prism 7.00.

### Immunofluorescence

For 2D immunofluorescence staining, cells were plated in a 96-well μclear plate (Greiner). Cells were fixed and permeabilized 72 h after plating by incubation with 1% formaldehyde and 0.1% Triton X-100 in PBS and blocked with 0.5% w/v BSA in PBS. Cells were incubated with 1:10,000 Hoechst 33258 combined with 1:2000 rhodamine-phalloidin (Molecular Probes, R415) for 1 h at room temperature. Cells were imaged with a Nikon Eclipse Ti microscope and 20 × oil objective.

### Hierarchical clustering and pathway analysis

Over-representation analysis was performed using Consensus PathDB^65^ using KEGG and Reactome databases. Ranked gene set enrichment analysis (GSEA) was performed on the full ranked gene lists^66^. T-sne plots were generated using the tsne R package. Hierarchical clusterings were generated using the pheatmap R package, based on complete linkage and Euclidean distance or correlations as described in the figure legend.

### Data retrieval and normalization

Normalized total gene expression data from The Cancer Genome Atlas (TCGA) were obtained by using the TCGA Assembler R package^67^ after the new release in January 2017. Normalized reads were log2 transformed before further analyses were performed. For calculating spearman correlations between the TCGA and cell line RNA sequencing data, log2 normalized data were used for both datasets. Microarray data of primary tumors of 867 untreated patients (MA-867 dataset) used to calculate hazard ratios was previously published and publicly available (GSE2034, GSE5327, GSE2990, GSE7390 and GSE11121). Raw .cel files were downloaded, processed with fRMA and batch effects were corrected using ComBat.

### Ethical approval

This article does not contain any studies with human participants or animals performed by any of the authors.

## Supplementary Information

Supplementary Figure Legends.

Supplementary Figures.

Supplementary Table 1.

Supplementary Table 2.

Supplementary Table 3.

Supplementary Table 4.

Supplementary Table 5.

## Data Availability

The datasets generated during and/or analysed during the current study are available from the corresponding author on reasonable request. Raw sequencing data will become publicly available upon publication and can currently be obtained by request.

## References

[1] American Cancer Society. *Cancer Facts and Figures* (2017). 10.6004/jadpro.2020.11.2.1 PMC7848816 33532112

[2] Anders C, Carey L (2008). Understanding and treating triple-negative breast cancer. Oncology.

[3] Badve S (2011). Basal-like and triple-negative breast cancers: a critical review with an emphasis on the implications for pathologists and oncologists. Mod. Pathol..

[4] Fulford LG (2007). Basal-like grade III invasive ductal carcinoma of the breast: patterns of metastasis and long-term survival. Breast Cancer Res..

[5] Yao H (2017). Triple-negative breast cancer: is there a treatment on the horizon?. Oncotarget.

[6] Di Z (2014). Ultra high content image analysis and phenotype profiling of 3D cultured micro-tissues. PLoS ONE.

[7] Barretina J (2012). The cancer cell line encyclopedia enables predictive modeling of anticancer drug sensitivity. Nature.

[8] Gale M (2016). Screen-identified selective inhibitor of lysine demethylase 5A blocks cancer cell growth and drug resistance. Oncotarget.

[9] Garnett MJ (2012). Systematic identification of genomic markers of drug sensitivity in cancer cells. Nature.

[10] Jansen VM (2017). Kinome-wide RNA interference screen reveals a role for PDK1 in acquired resistance to CDK4/6 Inhibition in ER-positive breast cancer. Cancer Res..

[11] Thrane S (2015). A kinase inhibitor screen identifies Mcl-1 and Aurora kinase A as novel treatment targets in antiestrogen-resistant breast cancer cells. Oncogene.

[12] Vora SR (2014). CDK 4/6 Inhibitors Sensitize PIK3CA mutant breast cancer to PI3K inhibitors. Cancer Cell.

[13] Sachs N (2018). A living biobank of breast cancer organoids captures disease heterogeneity. Cell.

[14] Jabs J (2017). Screening drug effects in patient-derived cancer cells links organoid responses to genome alterations. Mol. Syst. Biol..

[15] Wong AH (2017). Drug screening of cancer cell lines and human primary tumors using droplet microfluidics. Sci. Rep..

[16] Bruna A (2016). A biobank of breast cancer explants with preserved intra-tumor heterogeneity to screen anticancer compounds. Cell.

[17] Fang Y, Eglen RM (2017). Three-dimensional cell cultures in drug discovery and development. SLAS Discov..

[18] Gao H (2015). High-throughput screening using patient-derived tumor xenografts to predict clinical trial drug response. Nat. Med..

[19] Hidalgo M (2011). A pilot clinical study of treatment guided by personalized tumorgrafts in patients with advanced cancer. Mol. Cancer Ther..

[20] Katt M, Placone A, Wong A, Xu Z, Searson P (2016). In vitro tumor models: advantages, disadvantages, variables, and selecting the right platform. Front. Bioeng. Biotechnol..

[21] Zanoni M (2016). 3D tumor spheroid models for in vitro therapeutic screening: a systematic approach to enhance the biological relevance of data obtained. Sci. Rep..

[22] Drost J, Clevers H (2018). Organoids in cancer research. Nat. Rev. Cancer.

[23] Van De Wetering M (2015). Prospective derivation of a living organoid biobank of colorectal cancer patients. Cell.

[24] Neve RM (2006). A collection of breast cancer cell lines for the study of functionally distinct cancer subtypes. Cancer Cell.

[25] Prat A (2013). Characterization of cell lines derived from breast cancers and normal mammary tissues for the study of the intrinsic molecular subtypes. Breast Cancer Res. Treat..

[26] Hollestelle A (2010). Distinct gene mutation profiles among luminal-type and basal-type breast cancer cell lines. Breast Cancer Res. Treat..

[27] Kao J (2009). Molecular profiling of breast cancer cell lines defines relevant tumor models and provides a resource for cancer gene discovery. PLoS ONE.

[28] Riaz M (2013). MiRNA expression profiling of 51 human breast cancer cell lines reveals subtype and driver mutation-specific miRNAs. Breast Cancer Res..

[29] Charafe-Jauffret E (2006). Gene expression profiling of breast cell lines identifies potential new basal markers. Oncogene.

[30] Kenny PA (2007). The morphologies of breast cancer cell lines in three-dimensional assays correlate with their profiles of gene expression. Mol. Oncol..

[31] Qi Y, Xu R (2018). Roles of PLODs in collagen synthesis and cancer progression. Front. Cell Dev. Biol..

[32] Hanahan D, Weinberg RA (2011). Hallmarks of cancer: the next generation. Cell.

[33] Mayer EL (2015). Targeting breast cancer with CDK inhibitors. Curr. Oncol. Rep..

[34] Tang A (2017). Aurora kinases: novel therapy targets in cancers. Oncotarget.

[35] Bhullar KS (2018). Kinase-targeted cancer therapies: progress, challenges and future directions. Mol. Cancer.

[36] Cicenas J (2016). The Aurora kinase inhibitors in cancer research and therapy. J. Cancer Res. Clin. Oncol..

[37] Deshaies RJ (2014). Proteotoxic crisis, the ubiquitin-proteasome system, and cancer therapy. BMC Biol..

[38] Adams J (2004). THE proteasome: a suitable antineoplastic target. Nat. Rev. Cancer.

[39] Sun Y (2011). Effects of an indolocarbazole-derived CDK4 inhibitor on breast cancer cells. J. Cancer.

[40] Choi JE (2015). Combined treatment with ABT-737 and VX-680 induces apoptosis in Bcl-2- and c-FLIP-overexpressing breast carcinoma cells. Oncol. Rep..

[41] Lamb R (2013). Cell cycle regulators cyclin D1 and CDK4/6 have estrogen receptor-dependent divergent functions in breast cancer migration and stem cell-like activity. Cell Cycle.

[42] Li J-P (2015). The investigational Aurora kinase A inhibitor alisertib (MLN8237) induces cell cycle G2/M arrest, apoptosis, and autophagy via p38 MAPK and Akt/mTOR signaling pathways in human breast cancer cells. Drug Des. Dev. Ther..

[43] Maiello MR (2015). EGFR and MEK blockade in triple negative breast cancer cells. J. Cell. Biochem..

[44] Zhang M (2014). Prognostic value of survivin and EGFR protein expression in triple-negative breast cancer (TNBC) patients. Targ. Oncol..

[45] Nakai K, Hung M, Yamaguchi H (2016). A perspective on anti-EGFR therapies targeting triple-negative breast cancer. Am. J. Cancer Res..

[46] Savage P, Blanchet-cohen A, Kleinman CL, Park M, Rogoussis J (2017). A targetable EGFR-dependent tumor-initiating program in breast cancer. Cell Rep..

[47] Ramaiahgari SC (2014). A 3D in vitro model of differentiated HepG2 cell spheroids with improved liver-like properties for repeated dose high-throughput toxicity studies. Arch. Toxicol..

[48] Liu K (2019). Evaluating cell lines as models for metastatic breast cancer through integrative analysis of genomic data. Nat. Commun..

[49] Stone HR, Morris JR (2014). DNA damage emergency: cellular garbage disposal to the rescue ?. Oncogene.

[50] Chen S (2010). Genome-wide siRNA screen for modulators of cell death induced by proteasome inhibitor Bortezomib. Cancer Res..

[51] Lovitt CJ, Shelper TB, Avery VM (2018). Doxorubicin resistance in breast cancer cells is mediated by extracellular matrix proteins. BMC Cancer.

[52] Breslin S, Driscoll LO (2016). The relevance of using 3D cell cultures, in addition to 2D monolayer cultures, when evaluating breast cancer drug sensitivity and resistance. Oncotarget.

[53] Gangadhara S, Smith C, Barrett-lee P, Hiscox S (2016). 3D culture of Her2+ breast cancer cells promotes AKT to MAPK switching and a loss of therapeutic response. BMC Cancer.

[54] Mclaughlin RP (2019). A kinase inhibitor screen identifies a dual cdc7/CDK9 inhibitor to sensitise triple-negative breast cancer to EGFR-targeted therapy. Breast Cancer Res..

[55] Whittle JR, Lewis MT, Lindeman GJ, Visvader JE (2015). Patient-derived xenograft models of breast cancer and their predictive power. Breast Cancer Res..

[56] Papapetrou EP (2016). Patient-derived induced pluripotent stem cells in cancer research and precision oncology. Nat. Med..

[57] Tsai, H., Trubelja, A., Shen, A. Q. & Bao, G. Tumour-on-a-chip: microfluidic models of tumour morphology, growth and microenvironment. *R. Soc. Publ.* (2017). 10.1098/rsif.2017.0137 PMC5493797 28637915

[58] Tia HT, Mohammad AA, Harr JC (2020). Identification of synergistic drug combinations using breast cancer patient-derived xenografts. Sci. Rep..

[59] Driehuis E, Kretzschmar K, Clevers H (2020). Establishment of patient-derived cancer organoids for drug-screening applications. Nat. Protoc..

[60] Liu C (2020). Translational oncology drug screening model meets cancer organoid technology. Transl. Oncol..

[61] Rodriguez AD (2020). Lab on a chip cancer drugs on intact tumor slices. Lab Chip.

[62] Love MI, Huber W, Anders S (2014). Moderated estimation of fold change and dispersion for RNA-seq data with DESeq2. Genome Biol..

[63] Vichai V, Kirtikara K (2006). Sulforhodamine B colorimetric assay for cytotoxicity screening. Nat. Protoc..

[64] Zhang Y (2011). Elevated insulin-like growth factor 1 receptor signaling induces antiestrogen resistance through the MAPK/ERK and PI3K/Akt signaling routes. Breast Cancer Res..

[65] Kamburov A, Wierling C, Lehrach H, Herwig R (2009). ConsensusPathDB—a database for integrating human functional interaction networks. Nucl. Acids Res..

[66] Subramanian A (2005). Gene set enrichment analysis: a knowledge-based approach for interpreting genome-wide expression profiles. Proc. Natl. Acad. Sci..

[67] Zhu Y, Qiu P, Ji Y (2014). TCGA-assembler: an open-source pipeline for TCGA data downloading, assembling and processing. Nat. Methods.

