# Supplementary material for: Differential reprogramming of breast cancer subtypes in 3D cultures and implications for sensitivity to targeted therapy
Source: Sci Rep. 2021 Mar 31;11:7259. doi: 10.1038/s41598-021-86664-7 (PMC8012355; doi:10.1038/s41598-021-86664-7)

Differential reprogramming of breast cancer subtypes in 3D cultures and implications for sensitivity to targeted therapy

Esmee Koedoot[1,5], Liesanne Wolters[1], Marcel Smid[2], Peter Stoilov[3], Gerhard A. Burger[1], Bram Herpers[4], Kuan Yan[4], Leo S. Price[4], John W.M. Martens[2], Sylvia E. Le Dévédec[1], Bob van de Water[1]

[1] Division of Drug Discovery and Safety, LACDR, Leiden University, the Netherlands

[2] Department of Medical Oncology and Cancer Genomics Netherlands, Erasmus MC Cancer Institute, Erasmus University Medical Center, Rotterdam, the Netherlands

[3] Department of Biochemistry and Cancer Institute, Robert C. Byrd Health Sciences Center, West Virginia University, Morgantown, USA

[4] OcellO, Leiden, The Netherlands.

[5] To whom correspondence should be addressed

[5]Address of correspondence:

Esmee Koedoot
Division of Drug Discovery and Safety
Leiden Academic Centre for Drug Research
Leiden University
2300 RA Leiden
The Netherlands
Tel: +31-71-5276223
Fax: +31-71-5274277
e.koedoot@lacdr.leidenuniv.nl

The authors declare no potential conflicts of interest.

Supplementary Figure 1

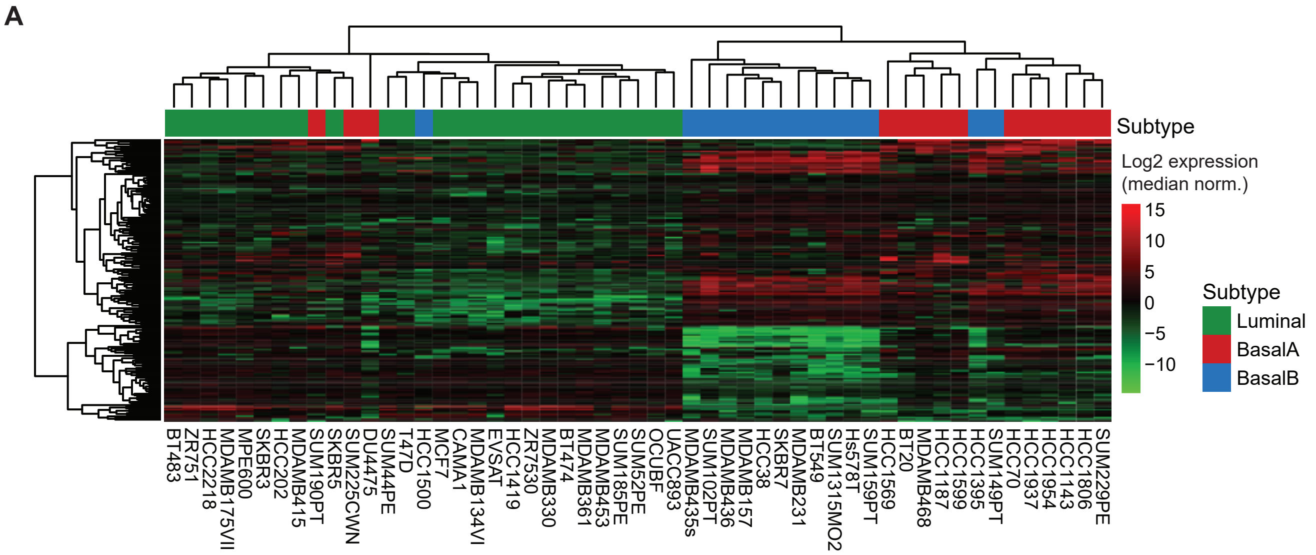

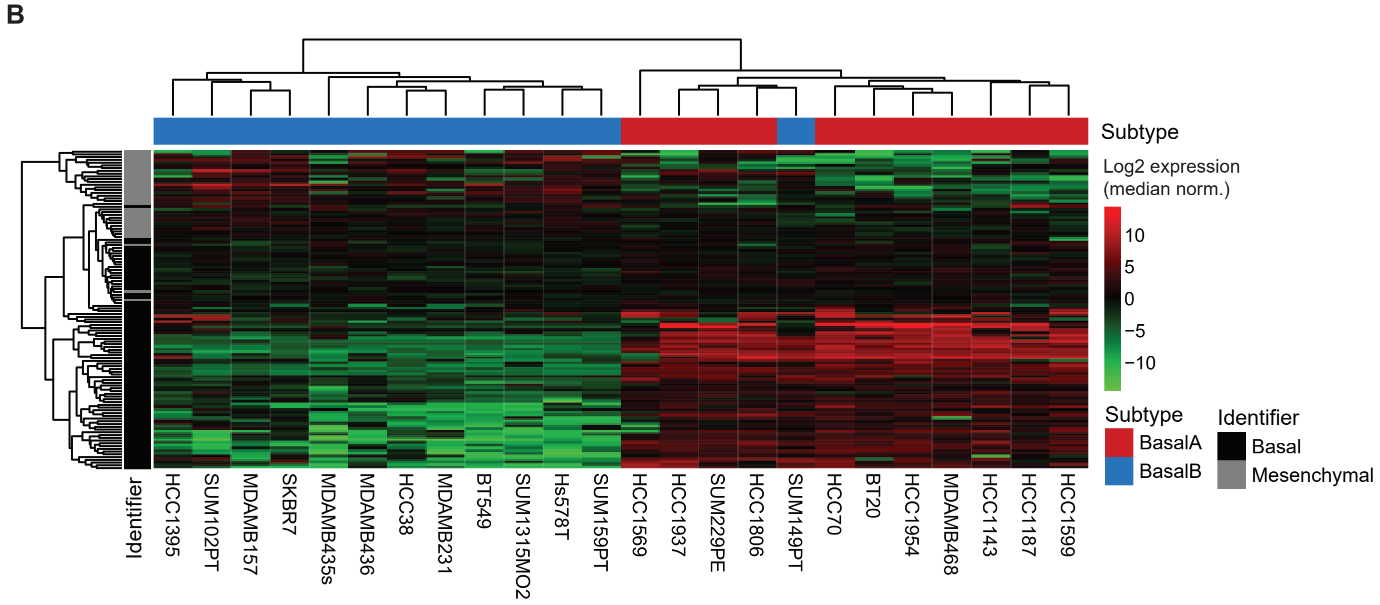

Supplementary Figure 2

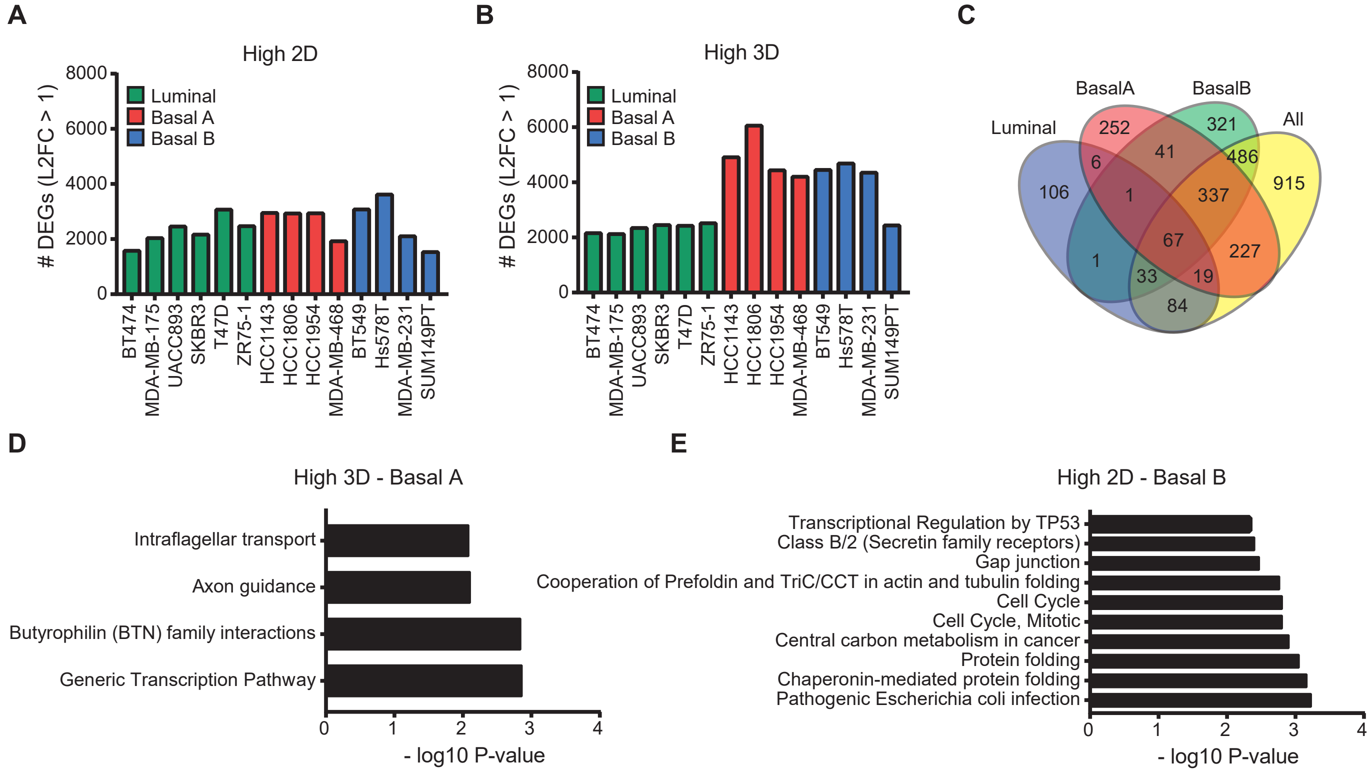

Supplementary Figure 3

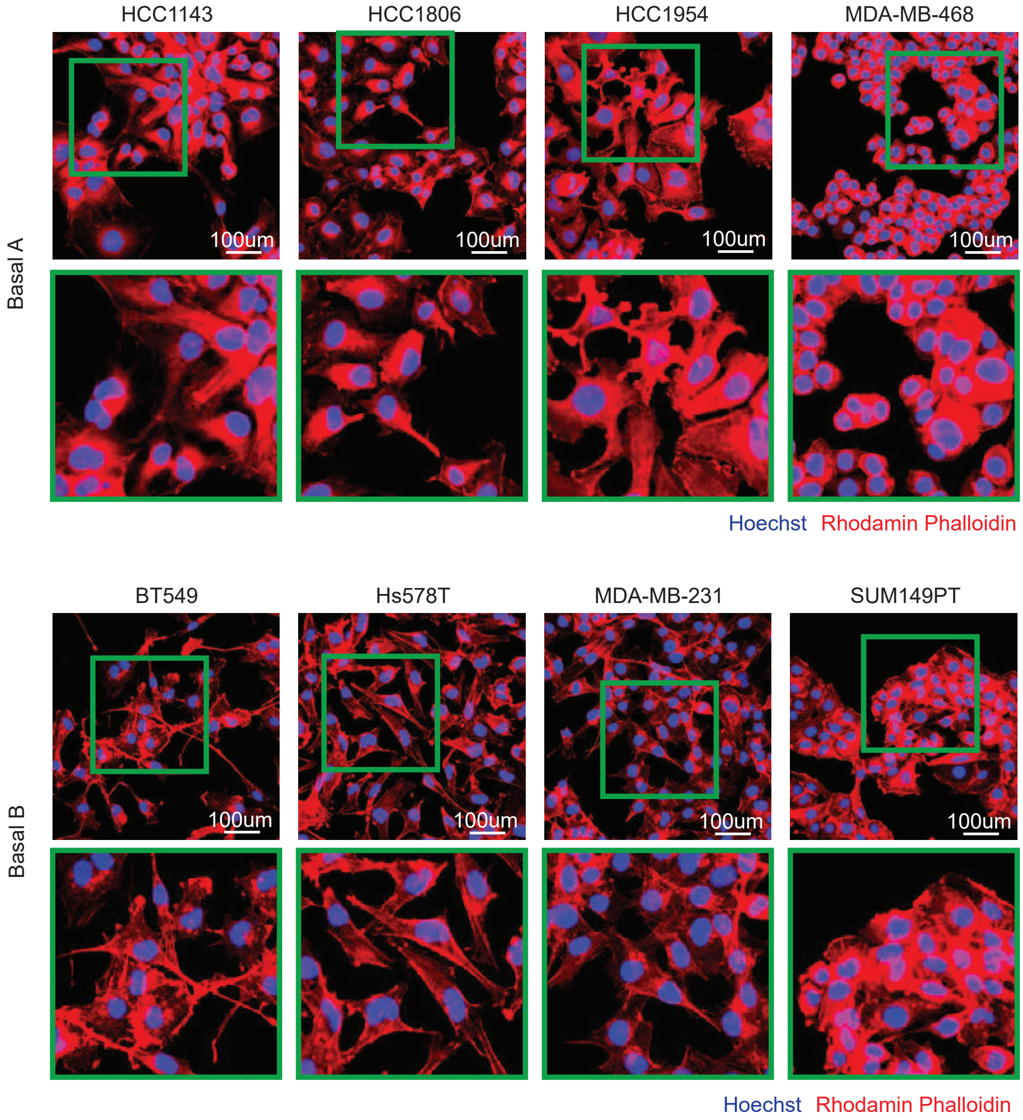

Supplementary Figure 4

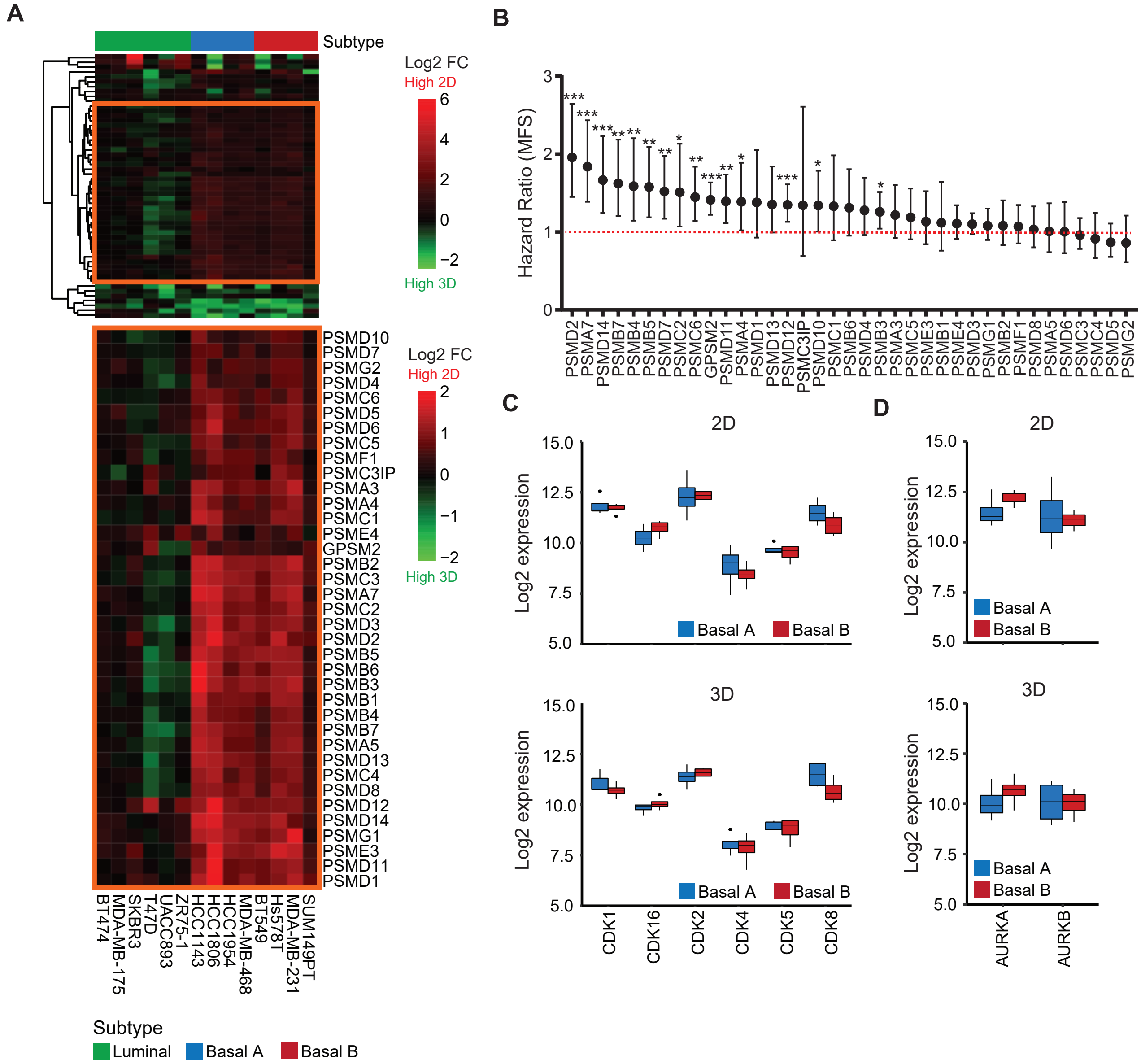

Supplementary Figure 5

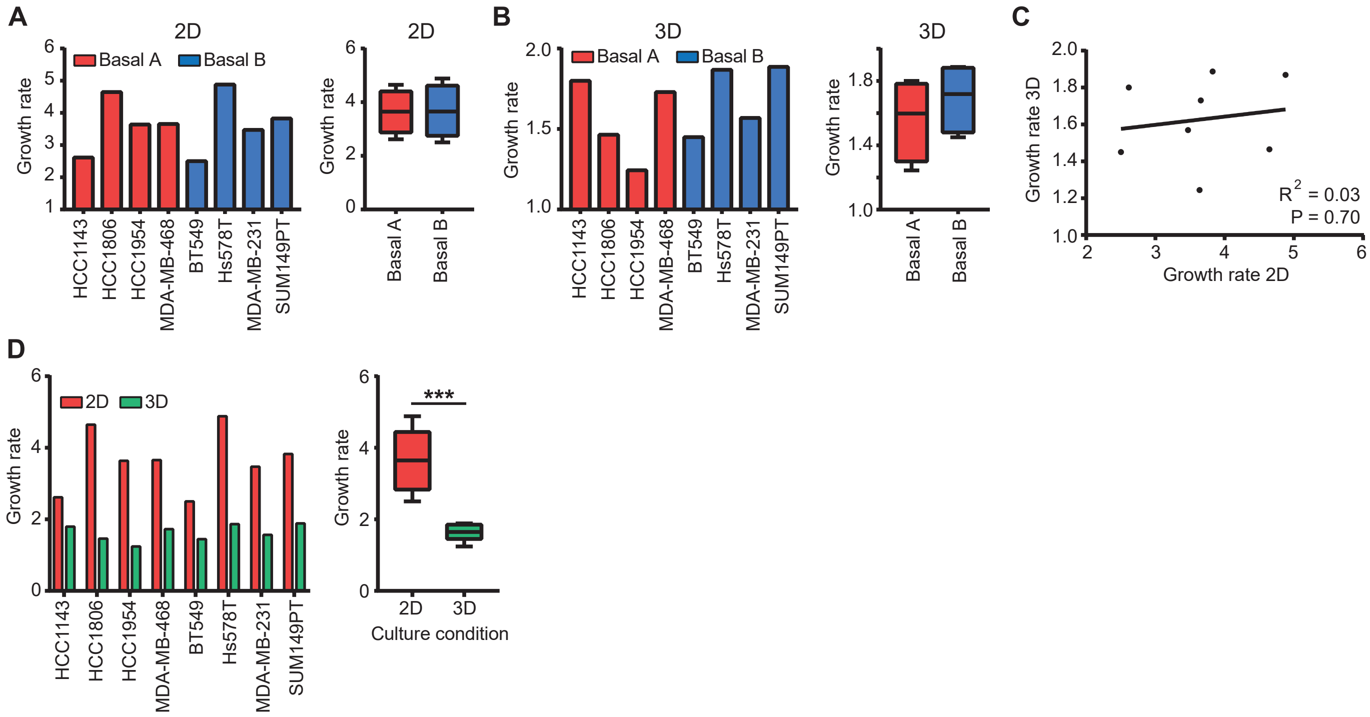

Supplementary Figure 6

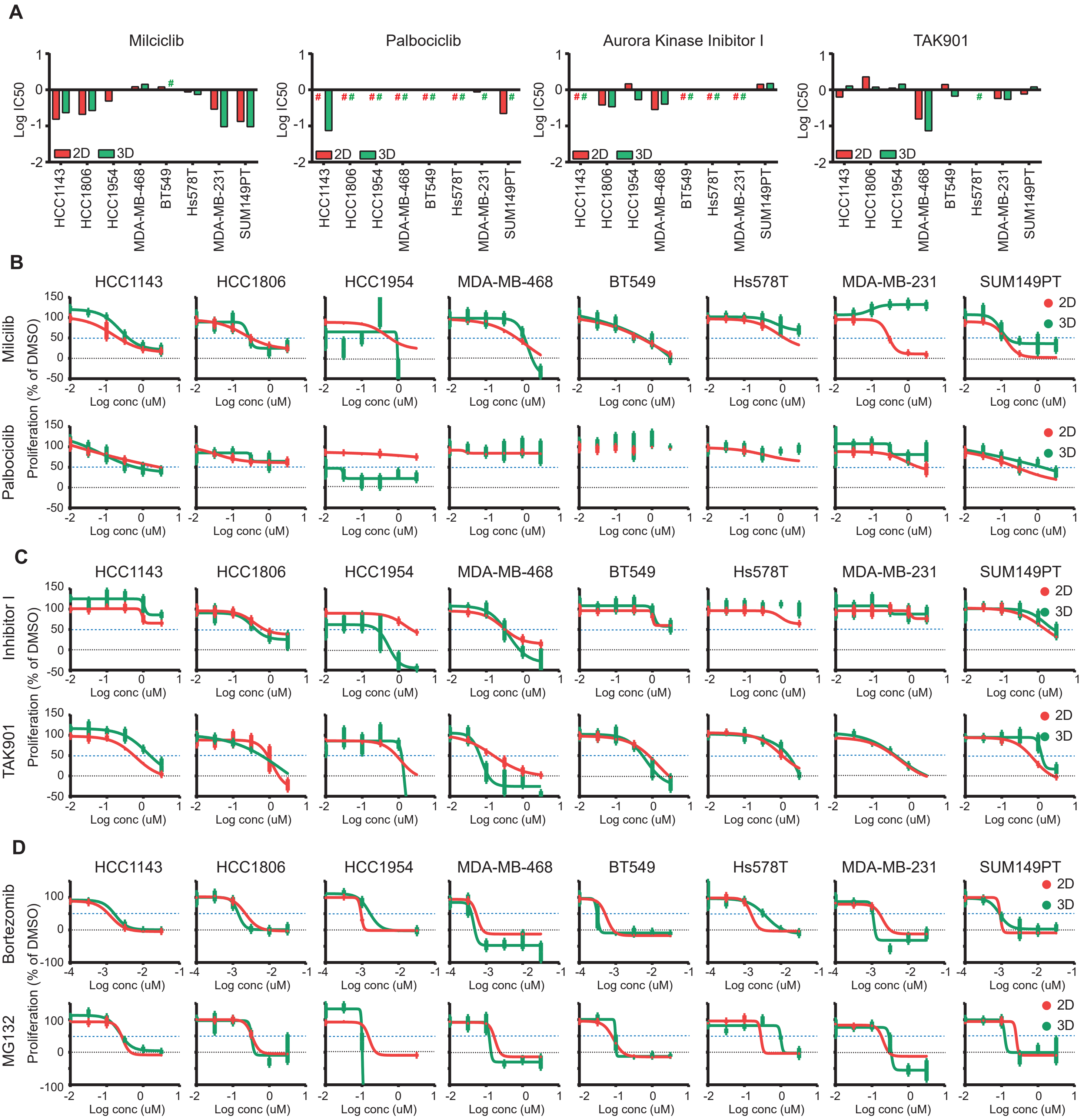

Supplementary Figure 7

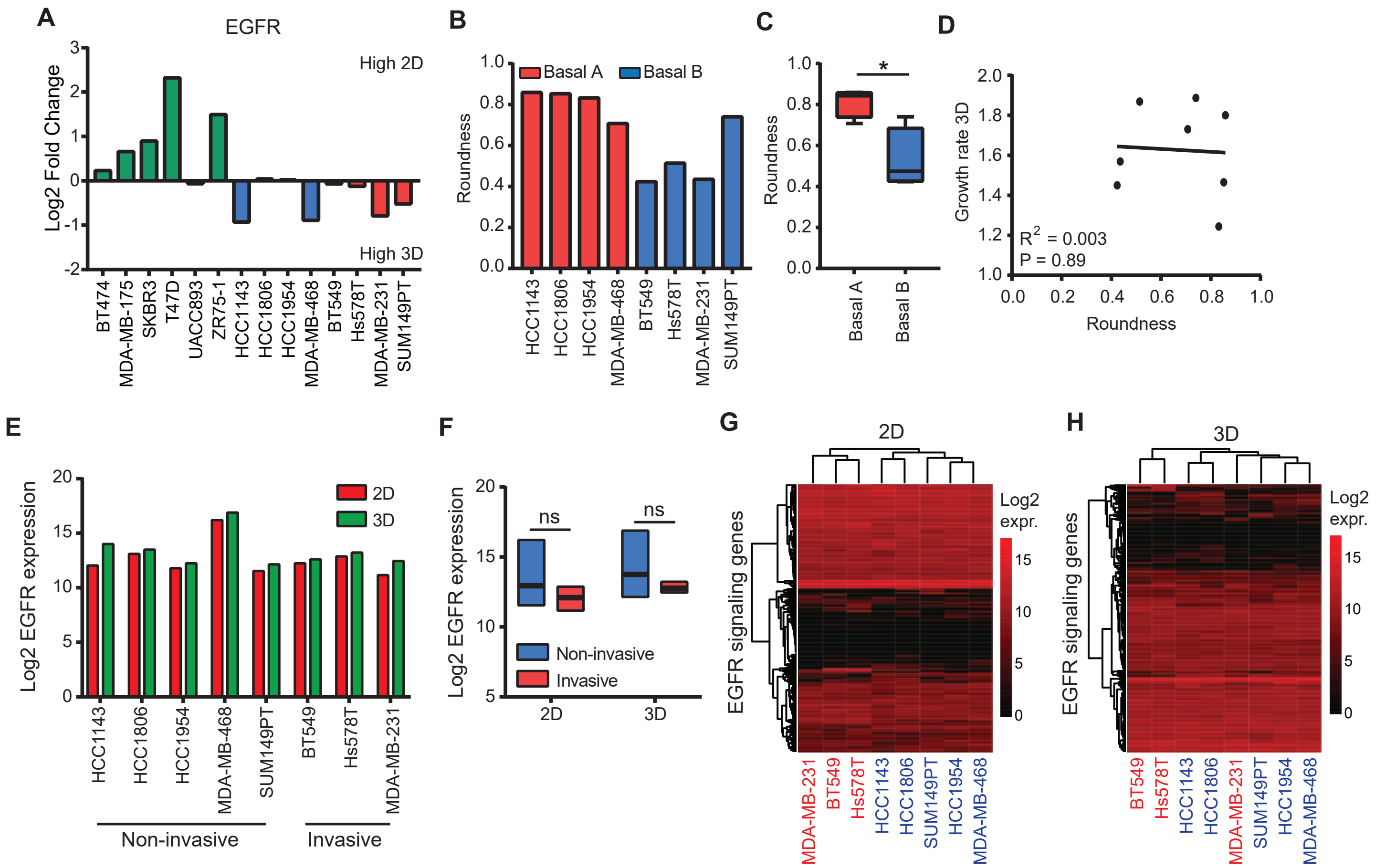

Supplementary Figure 8

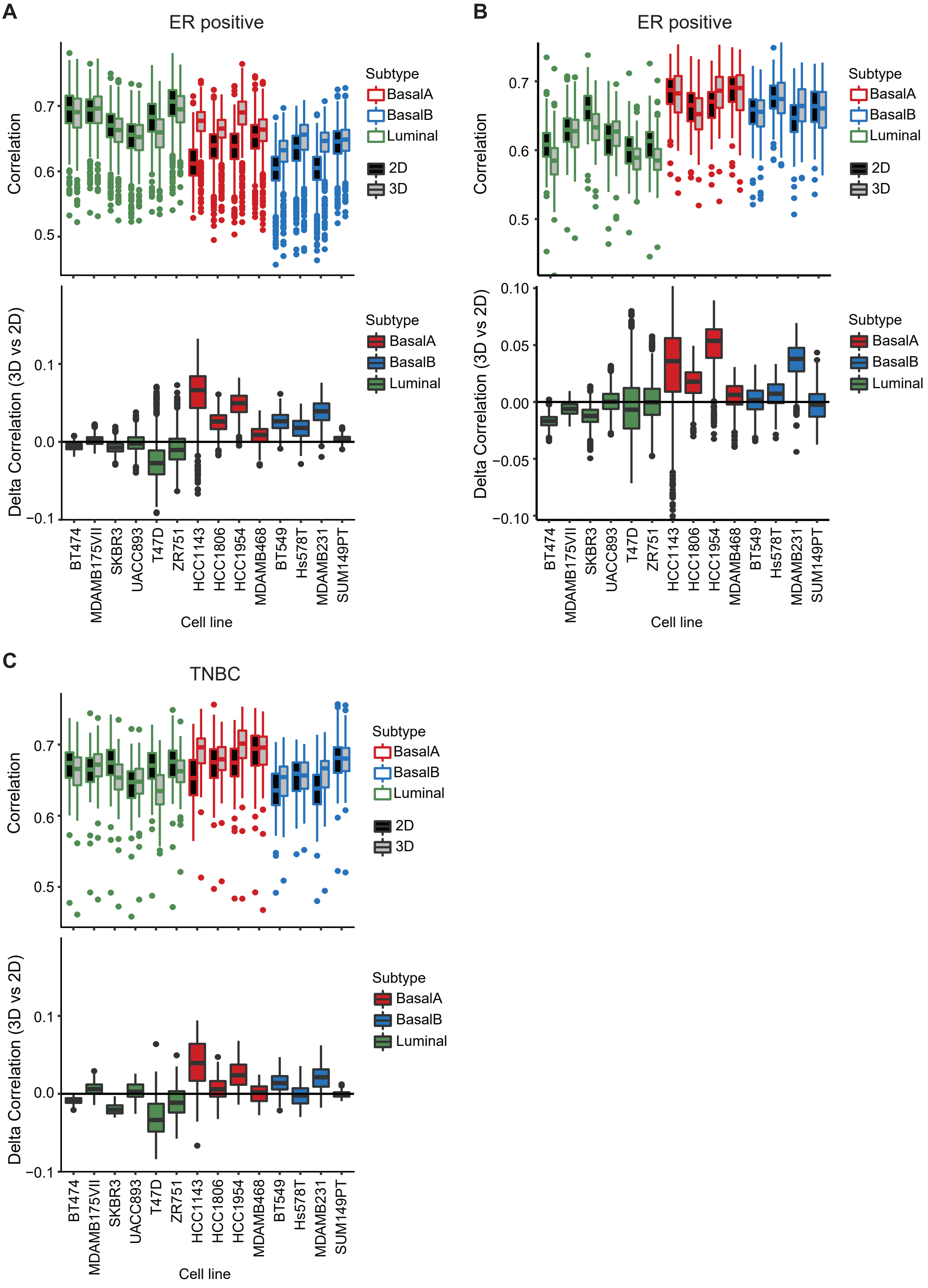

Supplementary Figure 9

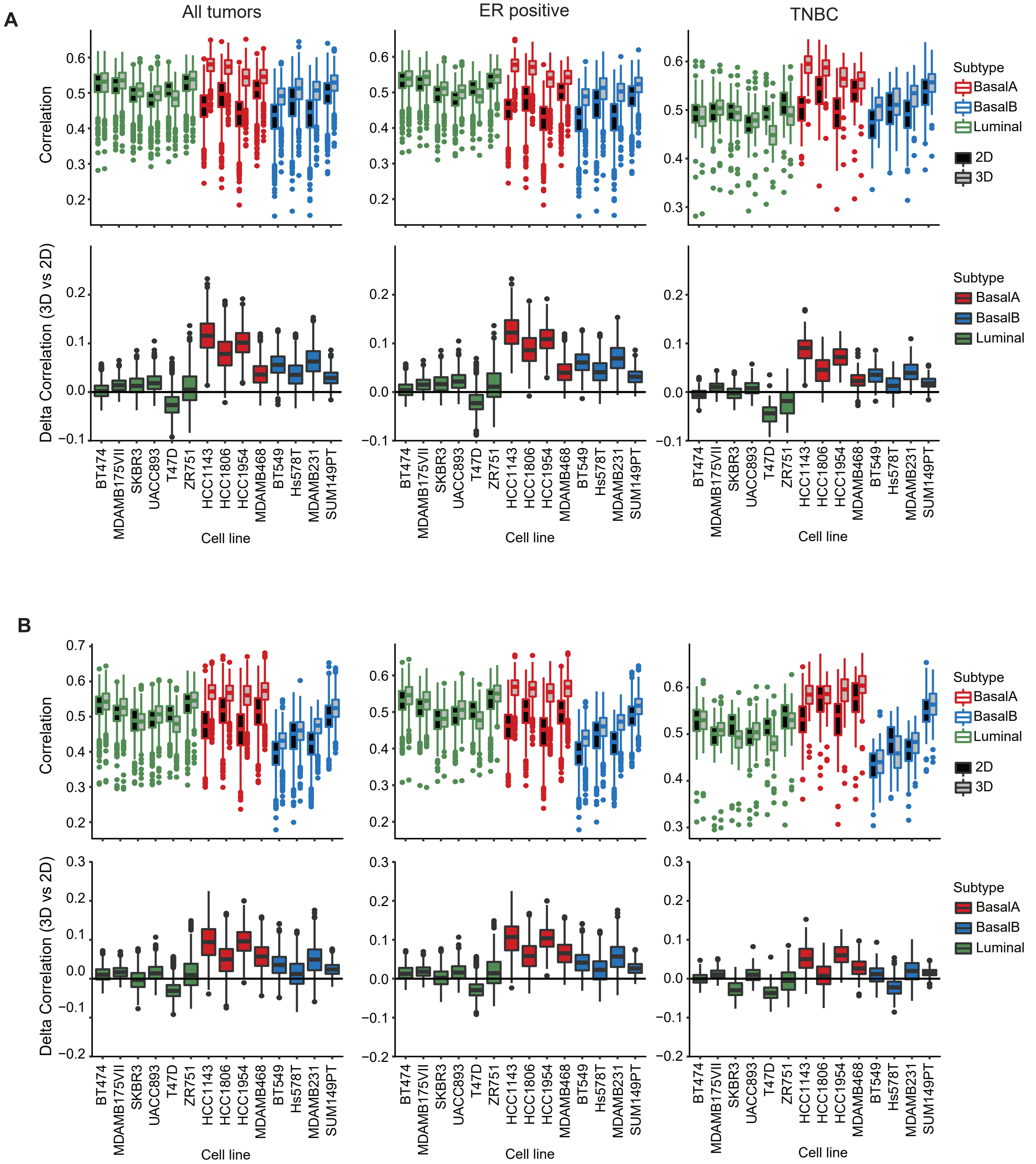

Supplementary Figure 10

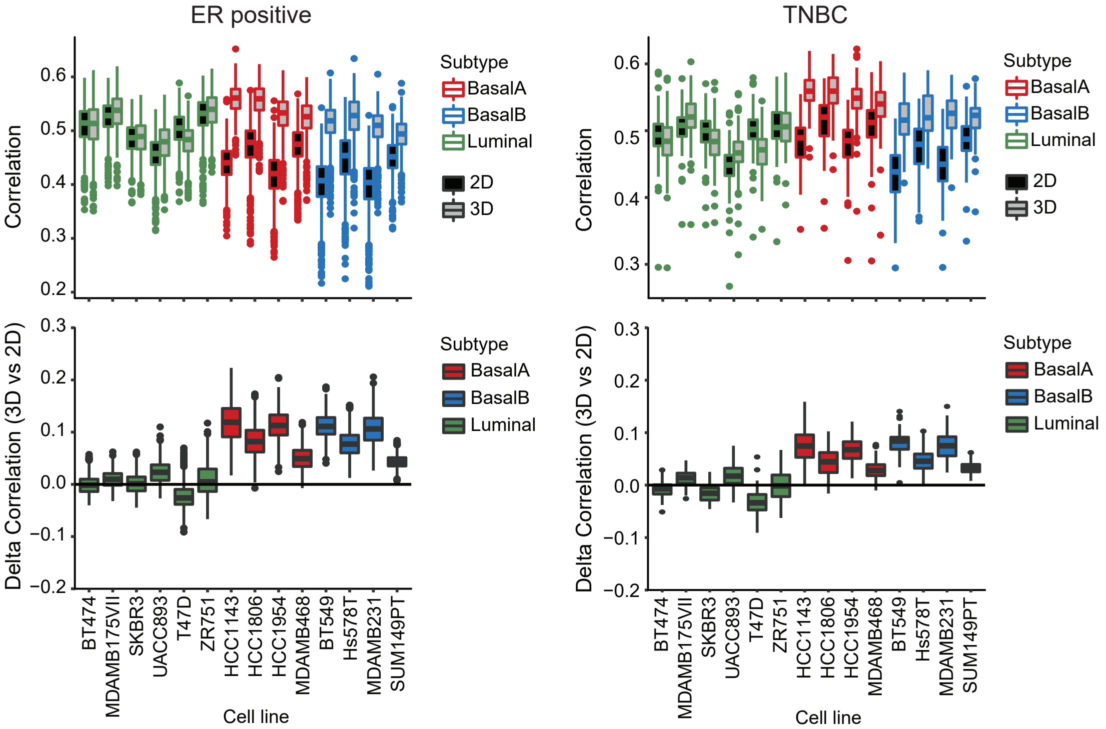

Supplementary Figure 11

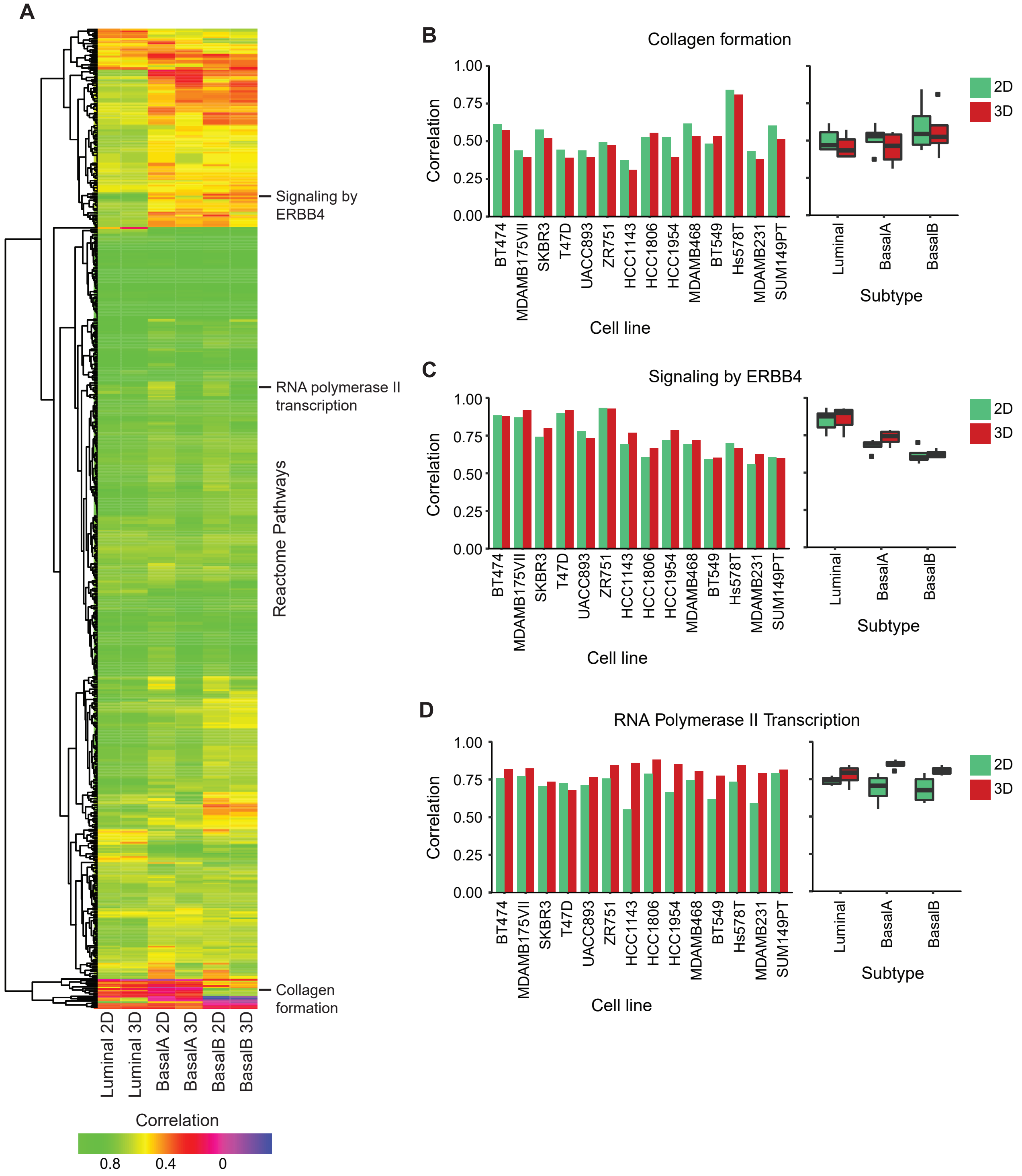

Supplement: Supplementary file 1 — Supplementary Figure Legends. [file 41598_2021_86664_MOESM1_ESM.pdf]
