# Supplementary material for: Differential reprogramming of breast cancer subtypes in 3D cultures and implications for sensitivity to targeted therapy
Source: Sci Rep. 2021 Mar 31;11:7259. doi: 10.1038/s41598-021-86664-7 (PMC8012355; doi:10.1038/s41598-021-86664-7)
Supplement: Supplementary file 2 — Supplementary Figures. [file 41598_2021_86664_MOESM2_ESM.docx]

*Supplementary Figure 1.* Subtype classifier expression in breast cancer cell lines.

(A) Hierarchical clustering (Euclidean distance, complete linkage) of log2 normalized RNA expression levels of basal-luminal identifiers^24^ in 2D cell cultures.

(B) Hierarchical clustering (Euclidean distance, complete linkage) of log2 normalized RNA expression levels of basal-mesenchymal identifiers^29^ in 2D cell cultures.

*Supplementary Figure 2.* Subtype specific differences in 2D and 3D breast cancer cultures.

(A) Number of DEGs higher expressed in 2D cultures per cell line (log2 fold change > 1).

(B) Number of DEGs higher expressed in 3D cultures per cell line (log2 fold change > 1).

(C) Overlap of significantly DEGs for different subtypes.

(D) Over-represented pathways in the high 3D DEGs in basal A cell lines. DEGs were determined by paired analysis of basal A cell lines, p < 0.01.

(E) Top 10 over-represented pathways in the high 2D DEGs in basal B cell lines. DEGs were determined by paired analysis of basal A cell lines, p < 0.01.

*Supplementary Figure 3*. Morphology of cell lines cultured in 2D.

*Supplementary Figure 4.* Role of the proteasome in breast cancer aggressiveness and metastasis formation.

(A) Hierarchical clustering (Euclidean distance, complete linkage) of log2 fold change of proteasomal factors comparing 2D to 3D cultures in all cell lines. Orange cluster is highlighted below.

(B) Hazard ratios and 95% CI for proteasomal factors higher expressed in 2D cultures (orange cluster in A).

(C) Log2 RNA expression of CDKs in basal A and basal B cell lines in 2D and 3D cultures. Median and interquartile range for 4 cell lines per subtype.

(D) Log2 RNA expression of aurora kinases in basal A and basal B cell lines in 2D and 3D cultures. Median and interquartile range for 4 cell lines per subtype.

*Supplementary Figure 5*. Growth rate of basal breast cancer cell lines in 2D and 3D cultures.

(A) Growth rate of basal breast cancer cell lines in 2D (amplification after 4 days of growth).

(B) Growth rate of basal breast cancer cell lines in 3D (amplification after 4 days of growth).

(C) Correlation between basal cell line growth rates in 2D and 3D culture conditions.

(D) Growth rates of basal breast cancer cell line in 2D and 3D.

*Supplementary Figure 6*. Drug sensitivity of cancer cell lines grown in 2D and 3D conditions.

(A) Log IC50 (uM) of CDK inhibitors milciclib, palbociclib and aurora kinase inhibitors aurora kinase inhibitor I and TAK901 in basal breast cancer cell lines 4 days after treatment.

(B) Dose-response curves of CDK inhibitors milciclib and palbociclib in basal breast cancer cell lines after 4 days of treatment.

(C) Dose-response curves of aurora kinase inhibitors aurora kinase inhibitor I and TAK901 in basal breast cancer cell lines after 4 days of treatment.

(D) Dose-response curves of proteasome inhibitors bortezomib and MG132 in basal breast cancer cell lines after 4 days of treatment.

*Supplementary Figure 7.* EGFR signaling in 2D and 3D cultures.

(A) Log2 fold change in EGFR expression levels comparing 2D to 3D cultures in different breast cancer cell lines.

(B) Roundness of different cell lines cultured in 3D.

(C) Roundness of basal A and basal B cell lines cultured in 3D. Median and interquartile range of 4 cell lines per subtype.

(D) Correlation between the 3D growth rate and roundness of basal cell lines.

(E) Log2 normalized EGFR RNA expression levels in 2D and 3D cultures for different basal cell lines.

(F) Log2 normalized EGFR RNA expression levels in 2D and 3D cultures for non-invasive and invasive cell lines. Median and interquartile range of cell lines shown in E.

(G) Heatmap (Euclidean distance, complete linkage) of log2 RNA expression of genes in the EGFR signaling pathway in non-invasive (blue) and invasive (red) cell lines in 2D.

(H) Heatmap (Euclidean distance, complete linkage) of log2 RNA expression of genes in the EGFR signaling pathway in non-invasive (blue) and invasive (red) cell lines in 3D.

*Supplementary Figure 8.* Comparison of transcriptomes of 2D and 3D cell culture models with the primary tumor transcriptomes.

(A) Spearman correlation of log2 normalized RNA expression levels between cell lines and patient primary ER positive breast tumors (n = 981). Boxes represent median and interquartile range of all tumor correlations. All genes were included in the correlation calculations (top).

Difference in spearman correlation comparing 2D and 3D cell line cultures matched by patient tumor. All genes and ER positive tumors (n = 981) were included in the correlation calculations. Boxes represent median and interquartile range of the difference in spearman correlation for all tumors (bottom).

(B) Same as (A) but now only genes in the estrogen receptor-mediated signaling pathway were incorporated in the analysis.

(C) Spearman correlation of log2 normalized RNA expression levels between cell lines and patient primary TNBC breast tumors (n = 116). Boxes represent median and interquartile range of all tumor correlations. All genes were included in the correlation calculations (top).

Difference in spearman correlation comparing 2D and 3D cell line cultures matched by patient tumor. All genes and TNBC tumors (n = 116) were included in the correlation calculations. Boxes represent median and interquartile range of the difference in spearman correlation for all tumors (bottom).

*Supplementary Figure 9.* Correlation of DEG RNA expression levels between 2D and 3D cell culture models and patient-derived primary tumor samples.

(A) Spearman correlation of log2 normalized RNA expression levels of DEGs (paired analysis of all cell lines, p < 0.01) between cell lines and patient primary tumors. Boxes represent median and interquartile range of tumor correlations (top). Difference in spearman correlation of log2 normalized RNA expression levels of DEGs (paired analysis of all cell lines, p < 0.01) comparing 2D and 3D cell line cultures matched by patient tumor. Boxes represent median and interquartile range of the difference in Spearman correlation (bottom). All tumors (n = 1097, left), ER positive tumors (n = 981, middle) or TNBC tumors (n = 116, right) were used for the correlation calculations.

(B) Same as in A, but now for basal A DEGs (paired analysis of basal A cell lines, p < 0.01).

*Supplementary Figure 10.* Correlation of basal B DEG RNA expression levels between 2D and 3D cell culture and patient-derived primary tumors.

(A) Spearman correlation of log2 normalized RNA expression levels of basal B DEGs (paired analysis, p < 0.01) between cell lines and patient ER positive primary breast tumors (n = 981). Boxes represent median and interquartile range of tumor correlations. Only DEGs comparing 2D and 3D cultures for the basal B cell lines were included in the correlation calculations (top).

Difference in spearman correlation comparing 2D and 3D cell line cultures matched by patient tumor. Only DEGs comparing 2D and 3D cultures for the basal B cell lines and ER positive tumors were included in the correlation calculations (bottom). Boxes represent median and interquartile range of the difference in spearman correlation for all tumors.

(B) Same as in A, but now for TNBC primary tumors (n = 116).

*Supplementary Figure 11.* Pathway expression in cell lines compared to primary patient tumor material.

(A) Hierarchical clustering (Euclidean distance, complete linkage) of spearman correlation of Reactome pathways correlating median tumor expression to mean subtype-culture condition expression.

(B) Spearman correlation of the RNA expression levels of genes in the collagen formation pathway between cell line and primary tumor samples (n = 1097). The bar graph shows the mean patient correlation. The boxplot shows the median and interquartile range of the mean patient correlation for luminal (n = 6), basal A (n = 4) and basal B (n = 4) cell lines.

(C) Same as in B for genes in the ERBB4 signaling pathway.

(D) Same as in B for genes in the RNA polymerase II transcription pathway.
